# Central sleep apnea in a treatment-resistant migraine patient: a case report

**DOI:** 10.5935/1984-0063.20220048

**Published:** 2022

**Authors:** Zahra Parsapour, Erfan Torabi, Hamed Amirifard, Leila Emami

**Affiliations:** 1 Isfahan University of Medical Sciences, Child Growth and Development Research Center, Research Institute for Primordial Prevention of Non-Communicable Disease - Isfahan - Iran.; 2 Baharloo Hospital, Tehran University of Medical Sciences, Occupational Sleep Research Center - Tehran - Iran.; 3 Amir Alam Hospital, Tehran University of Medical Sciences, Otorhinolaryngology Research Center - Tehran - Iran.

**Keywords:** Headache, Sleep Apnea, Central, Migraine Disorders, Opium

## Abstract

**Introduction:**

Patients with migraine, who suffer from sleep apnea, whether obstructive or central, might lead to treatment-resistant headaches. In this study, we have reported a 42-year-old man with a confirmed treatment-resistant migraine headaches and hemiplegic attacks, who was referred to our sleep clinic for evaluation of sleep breathing problems.

**Case Report:**

The patient had recurrent attacks of migraine headaches with hemiplegic attacks. The patient had headache in the past 15 years that based on ICHD-3 criteria classified as hemiplegic migraine. The severity and recurrences of headache and hemiplegic attacks gradually increased for 1 year, before he referred to our sleep clinic that led to several hospital admissions. He had been evaluated for other causes of headache; it seems that other headache causes have been ruled out. Treatment with medication was not effective to abolish symptoms. He had a history of occasional snoring and his wife had witnessed multiple episodes of apnea and frequent awakening by feeling suffocation at sleep. The patient abused methadone since 2 years ago. Based on the findings in polysomnography, the patient was diagnosed with central sleep apneas. After titration, bi-level positive airway pressurespontaneous timed mode (BiPAP-ST) was prescribed for the patient. In one year of using BiPAPST the central apneas events were controlled, while the frequency of migraine headache decreased remarkably to one attack per month and the hemiplegic attacks resolved without any other change in his medical treatment or methadone use.

**Discussion:**

Patients with chronic headaches usually have insufficient sleep, sleep apnea and poor sleep quality, change in sleep architecture can be an introduced trigger for headache, furthermore in opium abusers, symptoms got intensified. Resolve exacerbating factors can reduce headache recurrence and severity.

**Conclusion:**

It is important to screen high-risk patients for possible sleep disorders such as apnea, especially in treatment resistant migraine cases. Also, we should assess analgesics or opioids abuses and a complete history for other risk factors of central sleep apnea.

## INTRODUCTION

Central sleep apnea (CSA) is characterized by recurrent cessation or attenuation of respiration during sleep resulting from a decline or absence of ventilatory effort^1^. CSA is associated with conditions including heart failure (HF), stroke (CVA), atrial fibrillation (AF), renal failure (CKD), and medications (e.g., long acting opiates)^2^. The prevalence of symptomatic CSA (i.e., CSA syndrome) appears to be higher among older adults, males, those with certain comorbid medical conditions such as heart failure, and patients who chronically use opioids^3^. CSA syndrome is more prevalent among men than women. In one population-based sample, the prevalence of CSA syndrome was 0.4 percent among men and zero percent among women, while central apneas were detected in 7.8 percent of men and only 0.3 percent of women^4^. Among opioid users, CSA is the most common form of sleep-disordered breathing (SDB), although mixed patterns may occur and the quality of the data is limited. Most studies are small and consist of referral populations. In two studies that reported on the relative distribution of central versus obstructive apneas among chronic opioid users, CSA was four to six times more common than obstructive sleep apnea (60 versus 10 percent and 30 versus 8 percent)^5^. The most consistently identified risk factors are low to low-normal body mass index and daily dose of opioid. In particular, daily doses greater than 200mg morphine dose equivalents appear to confer higher risk^6^. SDB has been observed in stable patients enrolled in methadone maintenance programs as well as those receiving high potency opioids for management of cancer-related pain^6,7^.

The circadian rhythm emphasizes a hypothalamic involvement on the other hand temporal correlation with sleep stages (REM) of migraine attacks has role in brainstem dysfunction, which these networks involved in sleep stages regulation^8^, furthermore these have impressions not only on the physiology of sleep-wake and pain transmission and modulation, but also their dysfunction can explain relationship of migraine and insomnia^9^. It has been a long time that we now the relationship between headache and sleep^10^. A recent study showed that there is a significant relationship between primary headaches including migraine and severe sleep disorders measured by two valid sleep questionnaires^11^. Those disorders often coexist, and this has led to know that migraineurs have worse sleep quality than non-migraineurs^12-14^. Patients suffers from poor sleep quality are mostly associated with increased frequency of attacks or chronified migraine^15^. Migraine can be the result of sleep disruption led to it, or both may be symptoms of another medical condition, or they might be two related phenomena with pathophysiological mechanisms in common^10^. There are some studies which approve the co-accidence of migraine and central apnea during sleep but as said it is rare, for example in the study of 185 people only 1 person had CSA^16^. In addition to all above, chronic use of opioids disrupt sleep architecture^17^ and can adversely effect on respiratory depression, so being aware of the risk of sleep-disordered breathing in chronic use of opioids is important too^18^.

## CASE REPORT

A 42-year-old man was referred from department of neurology to the sleep clinic for evaluation of possible sleep apnea. His wife reported witnessed apneic episodes at night frequently awakening by feeling suffocation at sleep. As his past medical history, he had experienced several hospitalizations at neurology department along with several hemiparetic migraine attacks. Attacks were almost in the mornings upon awakening the patient, which he was forced to use analgesics to to relief headache but the headache did not subside completely during day. At the time, he underwent several paraclinical tests including MRI and MRA, which did not have any notable pathological findings. He had undergone several different managements that only had been able to relieve the patient’s symptoms for a while (e.g., sertraline HCL/100mg/daily, valproate sodium/400mg/ daily, ASA/80mg/daily). He had a notable history of substance abuse for about 15 years who was referred to the addiction clinic for methadone maintenance therapy (MMT) (40mg/day) for a limited time but he continued abusing methadone illegally for two years while he was addicted to methadone and gradually raised the dosage.

When he came to sleep clinic, we got a complete history, in addition to the aforementioned recurring symptoms, he declared a history of occasional snoring and his chief complaint was witnessed several episodes of apnea that and frequent awakening by feeling suffocation during sleep. The patient experienced better sleep in a semi-sitting position. The patient did not mention any history of cardio vascular disorders.

In physical examination his Mallampati score was grade 2/4 and neck circumference was 36cm and BMI: 25.4m^2^/kg. He had an ABG test in which PH=7.44, PCO_2_=44.8mmHg, HCO_3_=29.2mmol and PO_2_ was 78mmHg and as he stated, chronic bronchitis caused by heavy cigarette smoking and substance abuse was diagnosed for him. Polysomnography (PSG) was performed with standard technique in our sleep laboratory.

**Figure 1:**
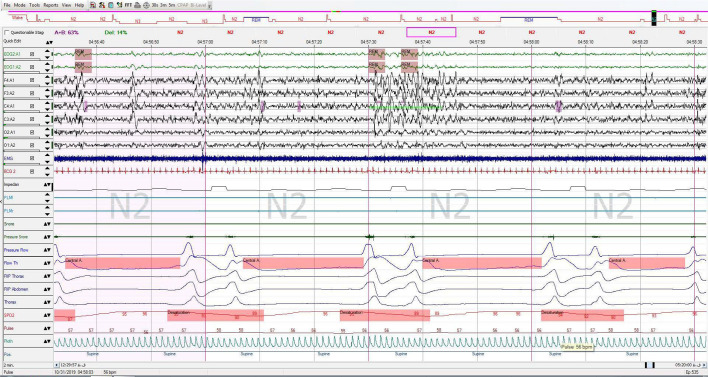
PSG study report (1/7).

**Figure 2:**
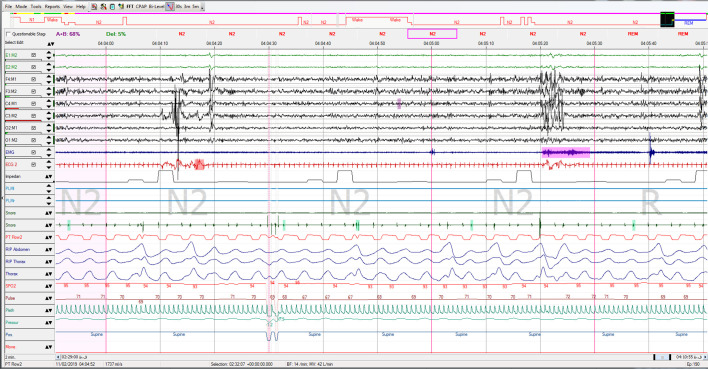
Images after improvement by BIPAP treatment that you requested.

**Figure 3:**
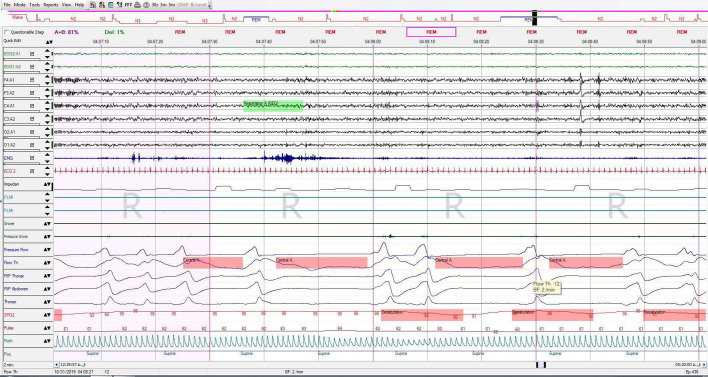
PSG study report (3/7).

**Figure 4:**
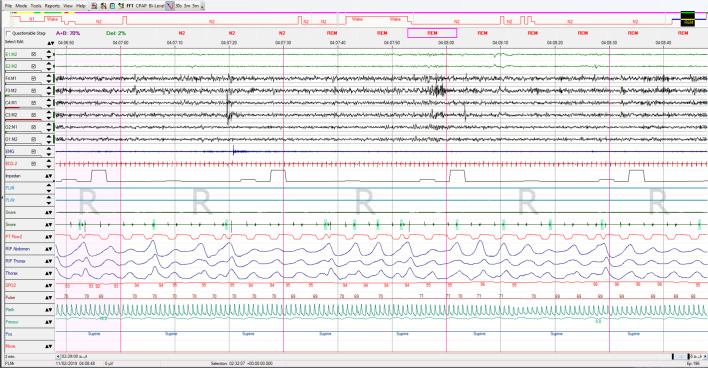
Images after improvement by BIPAP treatment that you requested.

**The results of full night standard PSG are as following (Figures 1-4):**

The patient had a total of 537 respiratory events including 504 central apnea events, which resulted in total respiratory disturbance index (RDI) of 115.6 respiratory events per hour and AHI: 115.6/hr and CAI: 108/hr and OBS: 6 (index: 1.3/ hr) of sleep. 7 obstructive apneas, only 1 mixed apnea and 26 obstructive hypopneas. Oxygen desaturation index (ODI) was 82.7/hr, 3% of desaturation event per hour of sleep and minimum oxygen desaturation was 83%.

Total sleep time was 4:38 hours in this study and the sleep efficiency was 96.2%, sleep latency was 7.5 minutes. Analysis of sleep stages distribution revealed that there was a decrease in REM stage, remarkable increase in stage N2 and decreased N3 stage, arousal index was 33 arousals per hour of sleep, the arousals mostly followed the desaturation and respiratory events. Electrocardiography reported normal sinus rhythm (NSR) with an average rate of 58bpm.

Based on the results of polysomnography and history of methadone abuse, the patient was diagnosed as central sleep apnea. Despite of suggestions of the literatures about using adaptive servo-ventilation for CSA, we had no choice but using BiPAP-ST because of limited equipment and patient financial conditions. We prescribed BiPAP-ST after titration study for the patient. The respiratory events were controlled optimally with IPAP:EPAP 17:11cmH_2_O and backup respiratory rate of 14 per minute.

One month after starting BiPAP-ST he came for a follow up visit at the sleep clinic, he did not complain of any headaches and hemiparetic attacks. He had an optimum compliance for the PAP device and he had used it for the total 30 days more than 4 hours per night. Average usage of device was 6 hours per night and AHI on the first month based on memory card was 20 respiratory events per hour of sleep. On his follow-up visit after 6 months, his compliance was optimum with an AHI of 15 respiratory events per hour of sleep.

He experienced a few episodes of headache with a frequency of one episode per months after 6 months, although he did not have any hemiparetic attacks. He was still compliant on using PAP device and AHI of the patient based on memory card was improved to 10 respiratory events per hour of sleep. When headache was controlled, we started tapering methadone for the patient but has not been successful until now.

## DISCUSSION

Central sleep apnea is one subtypes of sleep breathing disorders in which the brain “forgets” to send a signal to the breathing muscles. As the blood oxygen level reduces and blood carbon dioxide level increases, the brain is alerted. Then the brain send signal to the breathing muscles to function causing an “arousal.” The patients can have many arousals each hour. These prevent the patient from getting restorative sleep^19^. One of consequences of central apnea events is headache that might be secondary to arousals as well as hypoxia.

Andrijauskis et al.^20^, in 2019, worked on sleep disturbances of a random group of patients with active primary headaches (case group) and a control group. Patients with active primary headaches were further stratified into two groups: patients with migraine and patients with tension-type headache (TTH). They reported that patients with TTH were more likely to have insomnia (ISI score >7) than patients with migraine (75% vs. 50%, respectively) or controls (75% vs. 37.3%, respectively) (*p*=.002). Furthermore, TTH patients were more likely to have insufficient sleep (sleep efficiency <85%) (53.1%) than those with migraine (25%) or the control group (29.9%) (*p*=.025). They concluded that nearly all patients with TTH had poor sleep quality, and inadequate sleep can be introduced as a trigger factor for headache. This study can be a witness for our study that physicians should know how sleep disturbances play a significant role in control of migraine role in migraine or all types of headaches. In another recent study, Neau et al. (2002)^21^ found that out of 312 consecutive snoring patients who were referred for polysomnography study, 164 had sleep apnea syndrome.

Both the snoring group and the sleep apnea group had a high prevalence of headaches. They also found a high correlation between depression and snoring along with sleep apnea. It has been proposed that the desaturation of oxygen during an apnea event is the trigger for the cluster headaches (CH). The fact that oxygen can abort a CH supports this hypothesis. But treating sleep apnea does not necessarily prevent the CH^22^. Although there have been some reports that the OSA therapy helped the apnea, it is the author’s experience that patients with cluster in whom the OSA is controlled still have episodes with cluster. Buse et al. (2019)^23^ reported data from the Chronic Migraine Epidemiology and Outcomes (CaMEO) study to explore the relationship of migraine (episodic migraine (EM) and chronic migraine (CM)) with both sleep apnea, and measures of sleep quality. They stated that the increased risk of sleep apnea and breathing-related sleep problems was particularly high among men versus women and those with CM, higher BMI, and older age. Compared with respondents with EM, a larger proportion of those with CM were at “high-risk” for sleep apnea and reported poor sleep quality.

Substance abuse and the opium addiction in particular have increasingly turned into a societal and health burden especially in developing countries. Moreover, since 15 years ago and following the release of the American Academy of Pain Medicine and the American Pain Society’s joint statement^24^. In a study where 50 patients on MMT (methadone is a µ-opioid receptor agonist) were compared to 20 age- and sex-matched control subjects, findings showed 30% of the MMT subjects vs. no controls to have central sleep apnea (CSA)^25^. It has been hypothesized that the increased risk of CSA in MMT is linked to the imbalance between the central and peripheral chemoreceptors. In CSA, where the central chemoreceptors are inhibited, the peripheral receptors are enhanced and the stimulation of breathing following mild hypoxia would intermittently drive CO_2_ below the apnea threshold^7^. One of the side effects of methadone is headache and, it is another point, but is this patient the headache started 15 years ago and the patient abused opium secondary to severe untreated headache and his headache had hemiplegic migraine criteria. He was addicted to methadone since 2 years ago, while he started quitting addiction with methadone. Although he continued abusing methadone illegally and he was addicted to methadone since that time and he could not decrease methadone dosing until now, but his headache and hemiparetic attacks had controlled significantly after NIV therapy.

Bi-level positive airway pressure (BiPAP) and adaptive servo ventilation (ASV) with and without oxygen supplementation can be sought as a potential alternative. In a meta-analysis, BiPAP was shown to achieve elimination of central apneas in more than 60% of patients^26^. Central sleep apnea events can be treated with BiPAP-ST mode as well as adaptive servo ventilation. In our study, since the patient’s respiratory events were optimally controlled with BiPAP-ST, there was no need to ASV devices for the patient.

## CONCLUSION

Assessing sleep quality and screening for sleep apnea is valuable, especially among men and people with treatment resistant and severe migraine, CM, older age, and higher BMI. All people with migraine, particularly those with sleep disturbances such as insomnia, could benefit from being educated about behavioral sleep regulation and its likely value in the management of migraine. People with migraine who screen positive for sleep apnea should be referred for additional evaluation and possible positive airway pressure (PAP) treatment, including supportive education to ensure adequate adherence to PAP treatment. Also, perfect history taking can note special points to manage better, especially when patient abuse a substance which can trigger lots of symptoms like headaches and intensify complications of a disease like migraine which get resistant to treat. On the other hand, MMT for these patients must perform under close observations.
